# Mucus-Trap-Assisted Feeding Is a Common Strategy of the Small Mixoplanktonic *Prorocentrum pervagatum* and *P. cordatum* (Prorocentrales, Dinophyceae)

**DOI:** 10.3390/microorganisms11071730

**Published:** 2023-07-01

**Authors:** Urban Tillmann, Aditee Mitra, Kevin J. Flynn, Michaela E. Larsson

**Affiliations:** 1 Alfred Wegener Institute for Polar and Marine Research, Am Handelshafen 12, 27570 Bremerhaven, Germany; 2 School of Earth and Environmental Sciences, Cardiff University, Park Place, Cardiff CF10 3AT, UK; mitraa2@cardiff.ac.uk; 3 Plymouth Marine Laboratory, Plymouth PL1 3DH, UK; kjf@pml.ac.uk; 4 Aquatic Science Branch, Department of Water and Environmental Regulation, Joondalup, WA 6027, Australia; michaela.larsson@uts.edu.au

**Keywords:** microalgae, mixotrophy, mixoplankton, peduncle feeding, video microscopy, modelling

## Abstract

*Prorocentrum* comprises a diverse group of bloom-forming dinophytes with a worldwide distribution. Although photosynthetic, mixoplanktonic phagotrophy has also been described. Recently, the small *P.* cf. *balticum* was shown to use a remarkable feeding strategy by crafting globular mucus traps to capture and immobilize potential prey. Here we present evidence showing that two additional related species, the recently described *P. pervagatum* and the cosmopolitan bloom-forming *P. cordatum*, also produce large (80–120 µm) mucus traps supporting their mixoplanktonic activity. Prey are captured within the traps either through passive entanglement upon contact with the outside surface, or through active water movement created by rotating *Prorocentrum* cells eddying particles to the inside surface where trapped live prey cells became immobilized. Entrapment in mucus assisted deployment into the prey of a peduncle extruded from the apical area of the *Prorocentrum* cell. Phagotrophy by *P. pervagatum* supported faster growth compared to unfed controls and time series quantification of food vacuoles revealed ingestion rates of ca. 10–12 *Teleaulax* prey cells day^−1^. Model calculations show clear advantages of deploying a mucus trap for increasing prey encounter rates. This study demonstrates that the large size and immobilization properties of mucus traps successfully increase the availability of prey for small *Prorocentrum* species, whose peduncle feeding mode impedes consumption of actively moving prey, and that this strategy is common among certain clades of small planktonic *Prorocentrum* species.

## 1. Introduction

The genus *Prorocentrum* Ehrenberg comprises a diverse group of predominantly marine species with both benthic and planktonic representatives. Many species have a worldwide distribution and commonly form blooms in coastal systems. Cells of *Prorocentrum* are spheroid and laterally compressed, comprising two major thecal plates with a distinct sagittal suture; they lack a cingulum and sulcus. The two flagella emerge from the apical periflagellar pore (desmokont flagellation) [1,2], supporting a fast-swimming motion of a helical form with frequent changes in direction [3]. Traditionally viewed as being phytoplankton, like many other photoflagellates, some species of *Prorocentrum* are now recognized as being constitutive mixoplankton (CM)—protists that possess an innate capability to photosynthesize and can combine both phototrophy and phagotrophy synergistically [4]. Among planktonic *Prorocentrum*, *P. cordatum* (Ostenf.) J.D.Dodge [=*P. minimum* (Pavillard) Schiller], *P. micans* Ehrenberg, *P. redfieldii* Bursa (reported as *P. triestinum* J.Schiller), and *P. shikokuense* Hada (reported as *P. donghaiense* D.D.Lu) are reported as constitutive mixoplankton ([4], and references therein). The functional traits (i.e., phytoplankton vs. mixoplankton) of the other planktonic *Prorocentrum* remain unknown. Moreover, little is known about the details of the mechanisms employed for phagotrophy by the CM *Prorocentrum* species.

Dinoflagellate phagotrophic feeding mechanisms are typically grouped into three categories: (1) direct engulfment or phagocytosis which is more often observed in unarmoured species, (2) peduncular feeding (i.e., extruding a tube-like extension to pierce and extract the contents of prey cells) which is more common in armoured species, and (3) pallium feeding (i.e., extruding a membranous pseudopod which encloses a captured prey cell for “extracellular” digestion) which has only been recorded for heterotrophic dinoflagellate (i.e., not mixoplanktonic) species [5,6]. Until recently, it was believed that feeding in *Prorocentrum* species was via engulfment through the suture between the two thecal plates [7,8].

An exciting step forward in research on *Prorocentrum* feeding mechanisms was recently presented by Larsson et al. [9], who reported the novel use of a hollow mucus trap structure, termed a mucosphere, by a species of *Prorocentrum* provisionally identified as *P.* cf. *balticum*. This trap snares potential prey items, which are subsequently ingested by the dinoflagellate using a peduncle. The use of a peduncle requires that the predator–prey couple remains relatively stable during feeding. This state may be more easily attained by the predator immobilizing the prey using chemicals and/or with a physical intervention such as the deployment of mucus traps, as described in Larsson et al. [9]. This raises the question of the generality of such mucus traps used by other related mixoplanktonic *Prorocentrum*, their physiological advantages, and trophic implications.

The species *P.* cf. *balticum* form a distinct clade in rRNA sequence phylogenies which is embedded within a larger cluster of several small planktonic *Prorocentrum* [9,10]. This cluster of species include *P. cordatum* and taxa of the *P. shikokuense* group [10,11], which are both widely distributed and can be numerically abundant. Several new species within this group have also been recently described, including *P. pervagatum* Tillmann, Hoppenrath and Gottschling (=*P. criophilum* Gourvil and Gutiérrez-Rodríguez) [12], *P. spinulentum* Tillmann, Gottschling and Hoppenrath [10], and *P. thermophilum* F. Gómez, Tangcheng Li, Hu. Zhang & Senje Lin [13].

In this work we present microscopy video observations describing the deployment of mucus-trap structures and allied prey capture and feeding strategies of two species closely related to *P.* cf. *balticum*—the newly identified species *P. pervagatum* and the common, well-studied species, *P. cordatum.* Laboratory feeding experiments were performed to quantify growth and ingestion rates of *P. pervagatum* and to evaluate potential effects of turbulence on these processes. Finally, using a satiation-controlled encounter-based model, we also consider the fundamental differences and benefits between the prey encounter and capture rates by a solitary *Prorocentrum* cell versus a cell deploying a mucus trap.

## 2. Materials and Methods

### 2.1. Strains and Growth Conditions

Information on all algal strains and their respective growth conditions are compiled in Table 1. All strains of *Prorocentrum* were based on single cell isolation with micropipetting. All newly isolated strains were morphologically identified by light microscopy (LM) and Scanning Electron Microscopy (SEM), and confirmed by ribosomal gene sequencing of the 28S LSU and ITS marker genes ([12]; Tillmann and Larsson, unpublished). Strains were grown in controlled environment growth chambers (Friocell Evo, MMM Group, Müchen, Germany; or MIR 252, Sanyo Biomedical, Wood Dale, IL, USA) using K-based growth media [14] of different strengths (Table 1), which were modified by replacing the organic phosphorous source (β-glycerophosphate) with 3.62 µM di-sodium hydrogen phosphate (Na_2_HPO_4_). The prey items used were the cryptophyte *Teleaulax amphioxeia* (usually referred to as *Teleaulax* in the following), or occasionally *Rhodomonas salina* (Table 1).

### 2.2. Microscopy

Qualitative observation of mucus-trap formation and behaviour were performed for all *Prorocentrum* strains. Live observations of monocultures and mixtures of *Prorocentrum* strains isolated from the Labrador Sea, North Sea, and Black Sea, and cryptophyte prey, were performed in tissue-culture flasks or glass-bottom sedimentation chambers (Hydrobios, Kiel, Germany). These used a stereomicroscope (Olympus SZH-ILLD; Olympus, Hamburg, Germany) with dark-field illumination and/or an inverted microscope (Axiovert 200 M; Zeiss, Göttingen, Germany) equipped with epifluorescence and differential interference contrast optics. Cells were recorded using a digital video camera (Gryphax, Jenoptik; Jena, Germany) at full-HD resolution. Single frame micrographs were extracted using Corel Video Studio software (Version X8 pro; Corel; Ottawa, ON, Canada). Images of preserved cells (either Lugol, 1% final concentration, or formaldehyde, 1% final concentration) were taken with a digital camera (Axiocam MRc5; Zeiss). Swimming speeds of *P. pervagatum* were estimated using video frame-by-frame analyses of swimming individuals. Observations of the *Prorocentrum* strains from Western Australia were performed in glass-bottom 24 multi-well plates (Cellvis, Mountain View, CA, USA) using an inverted microscope (Leica DMI3000B, Leica, Wetzlar, Germany) equipped with differential interference contrast optics and a digital camera (BZ:1, Leica, Wetzlar, Germany).

### 2.3. Experiments

Quantitative experiments were performed with both *P. pervagatum* LP- strains (LP-D3 and LP-D10). To estimate ingestion rates, triplicate 15 mL culture volumes in 20 mL glass vials were prepared with each of the two *P. pervagatum* strains. The initial *Prorocentrum:Teleaulax* cell-ratio was approximately 1:20, with exponentially growing *Prorocentrum* (final abundance of 2000 cells mL^−1^) mixed with late-exponential-phase *Teleaulax amphioxeia* (final abundance of 40,000 cells mL^−1^). In addition, triplicate control vials of *Prorocentrum* strains without added prey were prepared. Vials were incubated under the previously described routine culture conditions. One mL was subsampled from all vials at T = 0, and then after 1, 2, 3, 4, 5, and 6 h, after gentle but thorough manual agitation of the vials. An additional triplicate set of 20 mL glass vials for each *P. pervagatum* strain mixed with *Teleaulax* was prepared, left undisturbed, and sampled after 6 h only. The two treatments of “sampled” and “undisturbed” were statistically compared using Student’s *t*-test. All subsamples were preserved with formaldehyde (1% final concentration) in small self-made sedimentation chambers. Samples were inspected at 640× magnification using epifluorescence under blue light excitation. The first 300 cells of *Prorocentrum* observed were scored for the presence and the number of food vacuoles. When the number of ingested prey was high, it was difficult to determine the number of food vacuoles and thus the highest category was set at ≥5 per *Prorocentrum* cell.

For long-term growth and ingestion rate estimation, triplicate 20 mL glass vials of strains LP-D10 and LP-D3 were set up at 1000 cells mL^−1^ initial abundance and supplied with 20,000 cells mL^−1^ *Teleaulax*. Additionally, triplicate controls of both *Prorocentrum* and *Teleaulax* monocultures were prepared, with all vials incubated under routine culture conditions. Subsamples to determine the cell abundance were taken at T = 0 and after 1, 2, 3, 4, and 7 days, preserved with Lugol’s solution (1% final concentration) in small sedimentation chambers, and counted with an inverted microscope. Subareas of the sedimentation chamber were counted to cover at least 400 cells of *Prorocentrum* and *Teleaulax* cells; where *Teleaulax* cell numbers were heavily depleted, subareas corresponding to 400 cells of the initial sample were counted. Each day in the morning after taking the subsample for cell enumeration, new *Teleaulax* were added to the mixtures to re-establish a *Prorocentrum*:*Teleaulax* cell-ratio of approximately 1:20. To attain the required additions, the cell abundance of the *Teleaulax* stock culture was estimated using a microscope counting chamber, and the required volume was added to each experimental vial. The dilution effects from these additions to experimental vials were taken into account when calculating the growth rates of *Prorocentrum* cells. The exponential growth rate μ (d^−1^) was calculated using linear regression of log-transformed cell number versus time for days 0–4 or 0–7 for strain LP-D10 and LP-D3, respectively. The growth rate was calculated for each replicate, and unfed and fed cultures were compared using Student’s *t*-tests.

For daily assessments of ingestion rates, a 1 mL subsample of each experimental vial was taken after 5 h following the addition of the *Teleaulax* prey. These subsamples were preserved with formaldehyde (1% final conc.) and the first 300 cells of *Prorocentrum* were scored for the number of food vacuoles using an inverted microscope under 640× magnification using epifluorescence with blue light excitation. For comparison, ingestion rates were determined based on day-to-day changes in *Teleaulax* cell abundance using the equations presented by Frost [15] and Heinbokel [16]. These calculations were performed either to include or exclude exponential growth of *Teleaulax* as determined with the *Teleaulax* control vials.

### 2.4. Modelling

To explore the implications of how deployment of a hollow mucus trap in which the *Prorocentrum* reside may enhance the competitive advantage of those species that utilise such structures, a model was developed from earlier work [17]. This “Satiation-Controlled Encounter-Based” (SCEB) model provides a mechanistic construct to explore predator–prey interactions as a function of allometry, motilities, encounter rates, and prey abundance under different levels of turbulence. SCEB was originally constructed to describe direct predation. Here SCEB was modified to include deployment of the mucus traps made by *Prorocentrum* to enable exploration of the implications of predation by the organism with a mucus trap. As it is not known whether all planktonic cells within a given *Prorocentrum* population need to form mucus traps to immobilize prey for feeding, the case where solitary *Prorocentrum* cells can engage in successful feeding upon a moving prey item was also considered.

Production and use of mucus traps by *Prorocentrum* have been observed only in static environments. The relationship between turbulence and mucus-trap production and their use has not been established. However, while turbulence is expected to increase predator–prey encounters, it may also affect the physical integrity of a mucus trap. For simplicity, three very low levels of turbulence were considered, assuming that these values do not affect the mucus-trap formation or functionality. Table 2 provides descriptions of the model parameters.

Movement of *Prorocentrum* cells appears to vary depending on the physiological state of the cell; for example, video observations show the swimming speed of *P. cordatum* cells to range between 110 and 200 µm s^−1^ (see Section 3, Results). In order to account for these changes in *Prorocentrum* motility, the scalar *sw_Mot_* (dl) is introduced in the SCEB description [17] of predator motility (Equation (1); *v_pred_*, m s^−1^) to modify the swimming speed around the allometrically-computed rate (where *ESD_pred_* = *Prorocentrum* diameter; µm). Thus, *sw_Mot_* values for a *Prorocentrum* cell of 13 µm diameter swimming at 110 µm s^−1^ and 200 µm s^−1^ are 0.7 and 1.3, respectively.

(1) $$v_{pred}={sw}_{Mot}\times \left(10^{-6}\times 38.542\times {{ESD}_{pred}}^{0.5424}\right)$$

Assuming the same propulsion effort is expended by both solitary and trap-producing cells, the motility of the trap propelled by the activity of a *Prorocentrum* cell within it would be governed by rules of hydrodynamics based on the relative size of the solitary cell (${ESD}_{pred}^{cell}$; µm) and that of the trap (${ESD}_{pred}^{trap}$; µm), and is defined as:

(2) $${Trap}_{mot}=\frac{{ESD}_{pred}^{cell}}{{ESD}_{pred}^{trap}}$$

The velocity of the trap ($v_{pred}^{trap}$; m s^−1^) can thence be calculated as:

(3) $$v_{pred}^{trap}={Trap}_{mot}\times v_{pred}$$

As ${ESD}_{pred}^{trap}$ > ${ESD}_{pred}^{cell}$, a *Prorocentrum* cell with a mucus trap would be expected to encounter many more prey items than one in a solitary (trap-less) state. The encounter rate also depends on the motilities of the two items (i.e., predator or trap, and the prey), and on turbulence (*w*). Accordingly, the original encounter rate description in SCEB would represent the prey encounter rate (${Enc}_{pred}^{cell}$, cell-specific prey predator^−1^ d^−1^) of a solitary *Prorocentrum* cell (Equation (4)). The modification of the encounter rate description for a *Prorocentrum* with a trap is given in Equation (5) (${Enc}_{pred}^{trap}$, prey trap^−1^ d^−1^).

(4) $${Enc}_{pred}^{cell}=\left(24\times 60\times 60\right)\times \pi {\left(\frac{{ESD}_{prey}}{2}+\frac{{ESD}_{pred}^{cell}}{2}\right)}^{2}\times N\times \left({v_{prey}}^{2}+3\times {v_{pred}}^{2}+4\times w^{2}\right)\times {\left({v_{pred}}^{2}+w^{2}\right)}^{-0.5}\times 3^{-1}$$

(5) $${Enc}_{pred}^{trap}=\left(24\times 60\times 60\right)\times \pi {\left(\frac{{ESD}_{prey}}{2}+\frac{{ESD}_{pred}^{trap}}{2}\right)}^{2}\times N\times \left({v_{pred}^{trap}}^{2}+3\times {v_{prey}}^{2}+4\times w^{2}\right)\times {\left({v_{prey}}^{2}+w^{2}\right)}^{-0.5}\times 3^{-1}$$

A predator cannot capture every prey item that it encounters; the proportion of prey captured is set by *Cap_pot_*. The rate of capture of prey cells by *Prorocentrum* in the solitary state (${Cap}_{pred}^{cell}$, prey predator^−1^ d^−1^) or with a trap (${Cap}_{pred}^{trap}$, prey trap^−1^ d^−1^) is described by Equation (6) and Equation (7), respectively, where the capture rate is described as function of the encounter rate (Equations (4) and (5), respectively), the likelihood of a predator capturing prey (*Cap_pot_*, dl), and the maximum number of prey that a predator can capture in a day (*Cap_max_*, prey cells d^−1^).

(6) $${Cap}_{pred}^{cell}=MIN\left({Cap}_{max},\;{Cap}_{pot}\times {Enc}_{pred}^{cell}\right)$$

(7) $${Cap}_{pred}^{trap}=MIN\left({{Cap}_{max},\;Cap}_{pot}\times {Enc}_{pred}^{trap}\right)$$

The maximum daily capture rates, *Cap_max_*, were calculated to provide the predator with sufficient N and P to support all their needs at the maximum growth rate, assuming the C:N:P of both prey and predator were the same. Considering a maximum predator growth rate of ca. 0.61 d^−1^ (see Section 3, Results), a predator to prey cell volume ratio of 4.3, and that retention of prey N and P will be less than 100% efficient, we have considered *Cap_max_* = 12 d^−1^. This is consistent with the measured ingestion rate of ca. 0.5 h^−1^ (=12 d^−1^, see Results). The exact number does not matter for the comparison between solitary and trap-equipped predators, assuming the ingestion rate into the *Prorocentrum* is the same.

In silico experiments were conducted to explore whether the presence of a trap affected encounter rates of prey by the predator and thence capture rates. Guided by the observational and in vivo experimental data (see Section 3, Results), the ESDs of the *Teleaulax* prey, the *Prorocentrum* solitary cell, and the mucus trap were set at 8 µm, 13 µm, and 80 µm, respectively. Calculations considered varying prey abundances in the environment (ranging from abundances likely encountered in nature to approaching those used in experiments, as 1–11,000 prey cells mL^−1^) under three different conditions of turbulence (0, 0.0005, and 0.001 m s^−1^).

To explore the effect of motility of the trap propelled by the activity of the *Prorocentrum* cell siphoning water through it, the model was configured with the mucus trap being non-motile and also motile. For all of the *Prorocentrum* cell configurations, we set the same values for *Cap_max_* and *Cap_pot_* in order to provide the same conditions to the cells with and without mucus traps. When motile, the scalar defining the velocity of the trap (*Trap_mot_*), calculated using Equation (2), was 0.1625. The likelihood of capturing a prey item was set at 20% according to Flynn and Mitra [17] for a cell–cell encounter situation; to account for the increased likelihood of capture when a prey is held immobile in a trap, likelihoods of 40%, 60%, and 95% were considered. Whether different volumes of a mucus trap, compared to the default volume (for cell of 80 µm), impacted capture rates was tested; mucus-trap volumes tested were of: (i) 0.5 × default (trap ESD = 63.5 µm), (ii) 2 × default (trap ESD = 101 µm), and the maximum observed trap ESD of 180 µm.

## 3. Results

Descriptions of behavioural aspects of the mucus-trap deployment and peduncular feeding are easiest to follow through reference to two extended Supplementary Video Files, one compiled for *P. pervagatum* strain LP-D10 (Video S1) and one for *P. cordatum* strain BS 4-A5 (Video S2).

### 3.1. Prorocentrum pervagatum

**Figure 1:**
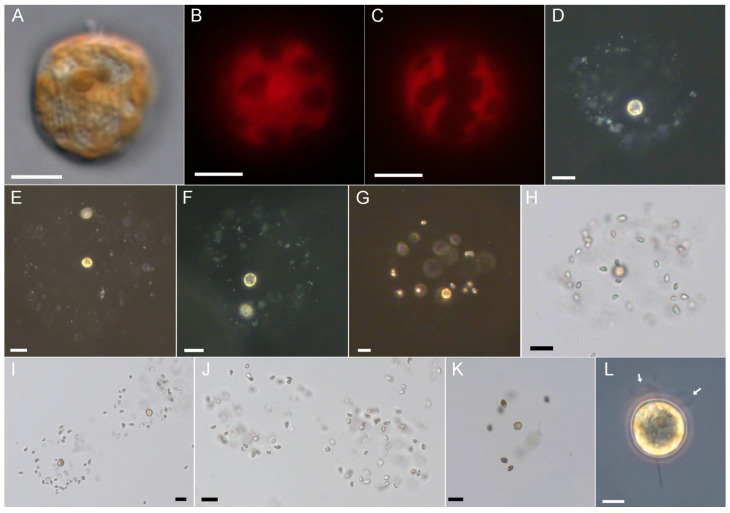
*Prorocentrum pervagatum*, strain LP-D10. (**A**) Cell in lateral view. (**B**,**C**) Epifluorescence view of chloroplasts (blue light excitation) of cells in lateral (**B**) or ventral (**C**) view. (**D**–**K**) Mucus traps in dark-field (**D**–**G**) or bright-field (**H**–**K**) illumination, loaded with bacteria (**D**–**F**), *Teleaulax amphioxeia* (**G**–**J**), or *Rhodomonas salina* (**K**). Image (**J**) shows two mucus traps without *Prorocentrum* cells. (**L**) Detailed view of a turning cell attached with the longitudinal flagellum. Note the double loop appearance of the transverse flagellum (arrow in (**L**)). Scale bars = 5 µm (**A**–**C**,**L**), or 20 µm (**D**–**K**).

*Prorocentrum pervagatum* [12] is a small (approximately 12–13 µm cell length) planktonic species with a round to oval outline in lateral view (Figure 1A) and two large and reticulate chloroplasts (Figure 1B,C). In monoculture, both swimming and drifting cells were observed in varying proportions (Video S1: 0:06 min). Swimming cells moved in straight or marginally wavy paths, at speeds of about 160 µm s^−1^ (range 110–200 µm s^−1^, n = 15). Drifting cells often occurred in pairs and appeared at low magnification as either still or with slowly rotating flagella movements.

In monoculture it was not feasible to identify if such drifting cells have mucus around them, but a mucus trap of drifting cells becomes visible when coated with particles of either bacteria or other microalgal cells (Figure 1D–K, Video S1: 1:00 min). The diameter of these globular or more irregularly shaped mucus traps were ca. 100 µm (mean 125 ± 20 µm, range 80–180 µm, n = 40) and there were no obvious differences between bacteria-loaded traps observed in xenic monoculture or traps with mixtures of *P. pervagatum* and *Teleaulax*. A *Prorocentrum* cell within a trap was typically located in the periphery. There were three different modes of movement of a *Prorocentrum* cell in a trap (Video S1: 2:20 min): (1) the most frequent involved the cell slowly rotating (one rotation took approximately 1–2.5 s) in a clockwise direction. Such cells were attached to the mucus trap with their longitudinal flagellum while creating a steady flow of the surrounding medium by the continuous beating of the transverse flagellum in a typical “double loop” arrangement (Figure 1L). (2) Cells could remain totally still without any visible beating of the flagella. (3) Occasionally, cells performed short and choppy “jumps” in various direction within the void of the trap interior.

A quantitative analysis of more than 4 hrs of video observation of 8 different mucus traps revealed that cells spent most time rotating (mean 68%), then jumping (mean 25%), with rotating and jumping periods lasting on average 48 s and 16 s, respectively, and the least time in still suspension, de facto drifting within the trap (8% of the total observation time), which lasted between 4 and 72 s. Cells switched motility modes approximately once per minute, although exceptionally short rotating and jumping periods were also observed. Rotating cells were always located in the periphery of the traps at one of relatively few fixed positions that were resumed after the intervening periods of jumping (Video S1: 3:55 min); in the following, these will be referred to as “rotating positions”. Phase contrast microscopy of *P. pervagatum* with added bacteria visualized the flow of particles by currents created by the rotating *Prorocentrum* cell (Video S1: 8:08 min), with major in- and outflows of particles at those areas of the mucus trap where the rotating *Prorocentrum* cell was positioned.

*Prorocentrum* could readily exit the mucus trap, leaving the empty mucoid structure laden with trapped prey (Figure 1J, Video S1: 4:49 min); vacated traps sank and accumulated at the bottom of the culture flasks. *Prorocentrum* cells leaving their traps were regularly observed when irritated or stressed by the fluorescence light source. Microscopy observation indicated that *Prorocentrum* cells likely leave the mucus trap at one of the “rotating positions”. This was most obvious in two instances (Video S1: 5:33 min), in which a freely swimming *Prorocentrum* cell had accidentally entered the mucus trap “owned” by another *Prorocentrum* cell. It took a while for such an “invading” *Prorocentrum* cell to exit the trap, which it did at one of the previously identified fixed positions of the rotating owner cell.

### 3.2. Interactions between P. pervagatum and the Cryptophyte Prey Teleaulax amphioxeia

In monoculture, *T. amphioxeia* cells were typically in constant motion and usually swam in slightly curved paths (Video S1: 9:18 min). However, when mixed with high densities (56 × 10^3^ cells mL^−1^) of *P. pervagatum*, *Teleaulax* cells were immediately affected, displaying a decreased motility compared to controls mixed with filtered sea water (Video S1: 9:40 min). *Teleaulax* cells, even when not immobilized in mucus traps, were clearly compromised, and displayed slow or no movement. 

The rotating behaviour of *Prorocentrum* cells inside a trap was repeatedly observed to eddy *Teleaulax* cells into the mucoid structure (Video S1: 10:09 min) where they would adhere to the inner surface. In addition to being eddied inside a trap by the rotating *Prorocentrum* cell, *Teleaulax* cells could become stuck to the outer surface of the mucus traps (Video S1: 13:50 min). *Teleaulax* cells approaching and eventually making contact with a mucus trap showed a suite of responses. These included responding with a number of rapid twitches, occasionally followed by short jumps, which either led to the cell becoming more entangled, or allowing a quick escape. Some cells were observed to perform rapid rotations on the spot, as if attached to a line. Such characteristic behavioural responses of *Teleaulax* were occasionally observed well before the cell encountered what appeared to be the outer edge of the trap, suggesting that there may be a far-reaching network of threads which is difficult to observe. The twitching and irregular movements could last for several seconds before the cell was eventually immobilized within the mucus. Regardless of the method of entanglement, *Teleaulax* cells held within the mucus trap were not dead but were instead fixed in position and exhibited brief tremor movements. Occasionally, however, *Teleaulax* cells held in mucus suddenly performed one or a series of short or more far-reaching jumps (up to 75 µm) of sufficient power to dislodge themselves from the mucus and escape.

**Figure 2:**
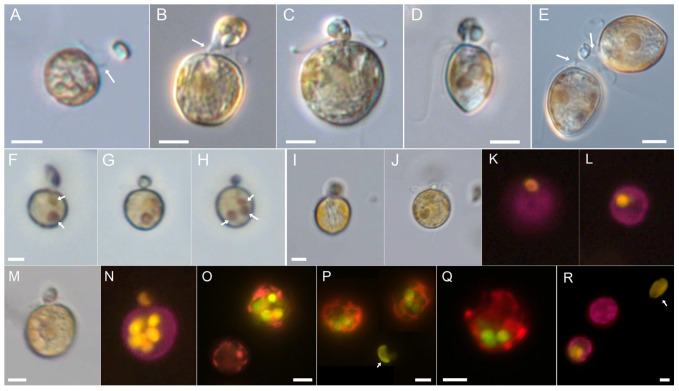
*Prorocentrum pervagatum* strain LP-D10 (**A**–**P**,**R**) or strain PM-01 (**Q**). Peduncle feeding in light (**A**–**J**,**M**) or epifluorescence (**K**,**L**,**N**–**R**) microscopy. (**A**) A cell just before attachment of the peduncle (arrow). (**B**–**D**) Different cells in lateral (**B**,**C**) and ventral (**D**) view with a *Teleaulax* prey cells attached via the peduncle in the apical position. Note the peduncle (arrow in (**B**)). (**E**) Two *P. pervagatum* cell having exhausted the contents of the same *Teleaulax* prey cell; note the peduncles (arrows) attached to the remains of the prey. (**F**–**H**) Time series of a *P. pervagatum* cell ingesting *Teleaulax*. Note the presence of two previous internal food vacuoles (arrows in **F**) before starting the new ingestion leading to a new vacuole, so that three internal food vacuoles are visible at a late stage of peduncular feeding (arrows in (**H**)). (**I**–**L**) The same cell in early (**I**,**K**) and late (**J**,**L**) stage of peduncular feeding as viewed in bright-field (**I**,**J**) or epifluorescence (**K**,**L**) light. One internal food vacuole is visible after ingestion. (**M**,**N**) The same peduncular feeding cell in lateral view under bright-field (**M**) or epifluorescence (**N**) view, note the yellow fluorescence of the externally attached *Teleaulax* cell and multiple internal food vacuoles. (**N**–**R**) Epifluorescence view of food vacuoles of *P. pervagatum* cells fed with *Teleaulax* (**N**–**Q**) or *Rhodomomas* (**I**). (**Q**) illustrates food uptake by the *P. pervagatum* type strain PM-01. Arrows in (**P**,**R**) indicate free *Teleaulax* and *Rhodomonas* cells, respectively. Scale bars = 5 µm.

Feeding events (Video S1: 21:02 min) were typically preceded by a series of jumps by the predator, as if it was scanning the mucus trap for immobilized prey. An actual feeding event was started by apical contact of the *Prorocentrum* cell with a prey cell and with attachment of the peduncle, which usually took several seconds and was, in most cases, successful only after a few attempts. Between attempts, the short peduncle was sometimes faintly visible (Figure 2A).

On occasion it was observed that a *Teleaulax* cell, apparently immobilized in a mucus trap, upon contact of the peduncle would make a quick jump out of range of the pursuing *Prorocentrum* cell. After a successful docking of the peduncle, the content of the prey cell was sucked into the predator (Figure 2B–D). A slow rotation of the prey cell was usually observed as the internal volume decreased; the entire feeding process varied in duration but lasted between 4 and 11 min (mean 6.6 ± 2.0 min, n = 20). Exceptionally, two *P. pervagatum* cells were observed simultaneously feeding upon the same *Teleaulax* prey cell (Figure 2E). During feeding, there were periods when *P*. *pervagatum* was completely motionless or moved back and forth in short, jerky movements of a few µm. At the end of feeding, one or two small granules (presumably of starch) remained and were finally shed as residue. During peduncle feeding, small food particles at times could be seen flowing inside of the *Prorocentrum* cell (Video S1: 25:20 and 25:31 min), and reddish coloured food vacuoles were visible using light microscopy (Figure 2F–H,J,M).

Ingested food was clearly visible inside the predator cell with fluorescence microscopy by its distinct yellow fluorescence (Figure 2L,N–R); the contents of each *Teleaulax* cell were ingested into a single distinct food vacuole (Figure 2F–L). The time between two subsequent feeding events of one *P. pervagatum* within a trap could be as short as 80 or 260 s as observed on two occasions. Alternatively, longer periods without ingestion events were also observed, between 30 and 40 min (n = 5) of *P. pervagatum* cells in a laden mucus trap. Feeding was confirmed for both LP-D10 and LP-D3 strains (Figure 2H,I) and for the *P. pervagatum* type strain PM-01 after mixing with *Teleaulax* (Figure 2Q). Additionally, for the more intensely studied strain LP-D10, mucus traps and food vacuoles were observed after mixing with the larger cryptophyte *Rhodomonas salina* (Figure 2R).

**Figure 3:**
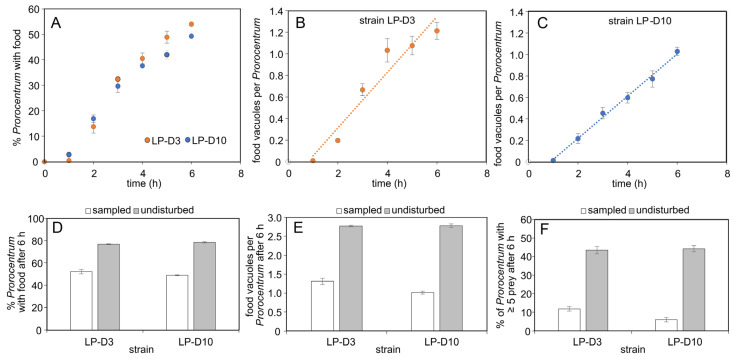
Short-term ingestion rate of *P. pervagatum* strains LP-D3 and LP-D10 when fed with the cryptophyte prey *Teleaulax amphioxeia*. (**A**) Time series of *Prorocentrum* cells with ingested food. (**B**,**C**) Increase in the average number of food vacuoles per *Prorocentrum* cell for strain LP-D3 (**B**) and LP-D10 (**C**). The dotted lines represent the linear regression. (**D**–**F**) Comparison of ingestion parameters (y-axis labels) after 6 h incubation for both strains from flasks sampled every hour (white bars) versus “undisturbed” flasks (grey bars) sampled after 6 h only. Data are treatment means (n = 3) ± 1SD. Differences between “sampled” and “undisturbed” (**D**–**F**) for both strains are significantly different (Student’s *t*-test, *p* < 0.001).

When incubated with *Teleaulax* as prey, the number of *P. pervagatum* cells with food vacuoles increased rapidly during the first 6 h (Figure 3A). The slope of the number of food vacuoles per *P. pervagatum* versus time revealed ingestion rates of 0.27 prey *Prorocentrum*^−1^ h^−1^ (strain LP-D3) or 0.19 prey *Prorocentrum*^−1^ h^−1^ (strain LP-D10) (Figure 3B,C). However, for both *P. pervagatum* strains there were significant differences between the flasks sampled every hour and those that were left undisturbed and sampled only after 6 h (Figure 3D–F). In these undisturbed samples the number of *P. pervagatum* cells with food vacuoles was significantly higher (Figure 3D) as were the number of food vacuoles per *P. pervagatum* (Figure 3E) and the number of cells with multiple (≥5) food vacuoles (Figure 3F). Observations of two *Prorocentrum* cells simultaneously feeding on one *Teleaulax* prey (Figure 2E, Video S1: 25:20 min) were very rare, and therefore, the bias on the ingestion rate calculation from such an event can be neglected.

**Figure 4:**
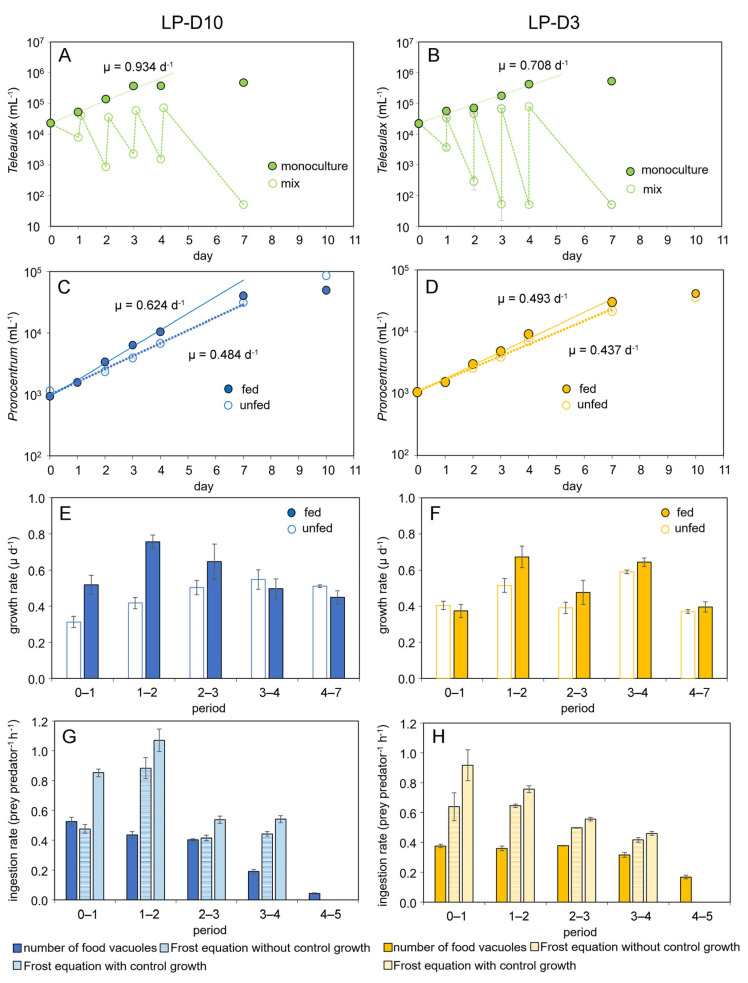
Long-term co-incubation of *P. pervagatum* strain LP-D10 (left panel) or LP-D3 (right panel) with *Teleaulax amphioxeia*. (**A**,**B**) Growth of *Teleaulax* in monoculture control (closed circles) and the concentration of *Teleaulax* cells in the mixed cultures (open circles). Note that new *Teleaulax* were added once per day to the mixed-culture flasks. (**C**,**D**) Growth curves of *P. pervagatum* in monoculture (unfed, open circles) and mixed culture (fed, closed circles). (**E**,**F**) Daily calculated exponential cell-specific growth rate µ (d^−1^) of *P. pervagatum* in monoculture (unfed, open circles) and mixed culture (fed, closed circles). (**G**,**H**) Ingestion rate, calculated using the number of food vacuoles as estimated 5 h after addition of new food (dark bars), or calculated using the Frost equations either considering simultaneous growth of the prey species in the mixed culture (light bars), or not (hatched bars). Data points represent mean ± 1SD (n = 3).

Long-term growth and ingestion of both *P. pervagatum* LP strains were measured for 10 days with daily addition of new food for the first 4 days. *Teleaulax* cells grew exponentially in the monoculture control, but the number of *Teleaulax* cells in the mixtures with *P. pervagatum* decreased substantially every day (Figure 4A,B). Exponential growth rates of *P. pervagatum* were significantly higher when fed *Teleaulax* as prey (Student’s *t*-test, *p* < 0.05) compared to monoculture, with strain LP-D10 having a greater difference than strain LP-D3 (Figure 4C,D), and these differences were mainly due to markedly higher growth during the first days of the experiments (Figure 4E,F). Ingestion rate as estimated using the number of food vacuoles 5 h after the addition of new *Teleaulax* cells was ca. 0.4–0.5 prey *Prorocentrum*^−1^ h^−1^ and slightly decreased at the end of the experiment (Figure 4G,H). Ingestion rates calculated from the decline of *Teleaulax* abundance were substantially higher, with rates not considering an exponential increase in food showing a better agreement with rate estimates based on food vacuoles (Figure 4G,H).

### 3.3. Prorocentrum cordatum

**Figure 5:**
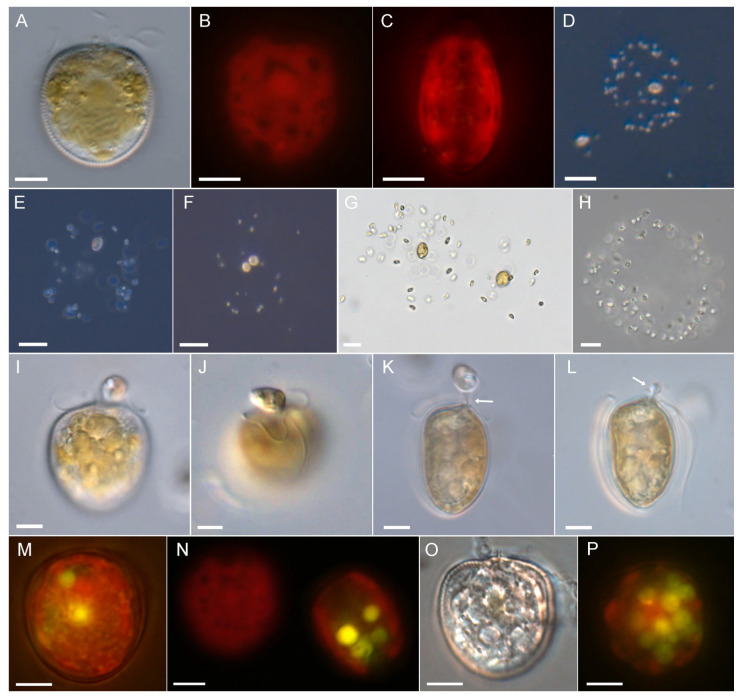
*Prorocentrum cordatum*, strain BS 4-A5. (**A**) Cell in lateral view. (**B**,**C**) Epifluorescence view of chloroplasts (blue light excitation) of cells in lateral (**B**) or ventral (**C**) view. (**D**–**H**). Mucus traps in dark-field (**D**,**E,F**) or bright-field (**G**,**H**) illumination, loaded with *Teleaulax amphioxeia*. (**I**–**P**) Peduncular feeding in light (**I**–**L**,**O**) or epifluorescence (**M**,**N**,**P**) microscopy. (**I**–**K**) Different cells in lateral (**I**), apical (**J**), or ventral (**K**) view with a *Teleaulax* prey cell attached in the apical position (arrow in (**K**) points to the extruded peduncle). (**L**) The same cell as in (**K**) shortly after feeding where the extruded peduncle is clearly visible (arrow). (**M**–**P**) Presence of food vacuoles after feeding on *Teleaulax*. (**O**,**P**) The same formalin-preserved cell in bright-field and epifluorescence view, displaying multiple food vacuoles. Scale bars = 50 µm (**D**–**F**), 20 µm (**G**,**H**), or 5 µm (**A**–**C**,**I**–**P**).

Food vacuoles within *P. cordatum* were observed for all three Black Sea strains after mixing with *Teleaulax*. Likewise, all four *P. cordatum* strains from Western Australia were observed to produce mucus traps and to consume *Rhodomonas salina* by peduncle feeding (Figure S1). Extended video observations using the *P. cordatum* strain BS 4-A5 (Video S2) revealed details of mucus traps, peduncular feeding, and similar modes of behaviour to *P. pervagatum*. Cells of *P. cordatum* strain BS 4-A5 were laterally compressed and slightly larger (ca. 18 µm in length) than *P. pervagatum* cells (ca. 13 µm; this roughly correspond to a 2-fold larger volume of *P. cordatum*) but similarly had two reticulated chloroplasts (Figure 5A–C).

In monoculture there were swimming cells, but at times a very high proportion of motionless suspended cells occurred, mostly as pairs of cells (Video S2; 0:07 min). Mucus traps were visible when *P. cordatum* cells were mixed with *Teleaulax* (Figure 5D–G) and were approximately 120 µm in diameter (120 ± 20 µm, range 70–165 µm, n = 23), with some observed without *Prorocentrum* cells but densely laden with prey cells (Figure 5H). Occasionally there were two *P. cordatum* cells in one trap (Video S2: 3:02 min).

*Prorocentrum cordatum* cells in traps exhibited the same jumping and rotating behaviour as described for *P. pervagatum* (Video S2: 3:25 min). Likewise, behavioural responses of *Teleaulax* cells approaching a trap of *P. cordatum* were similar to those described for mixtures of *Teleaulax* with *P. pervagatum* cells (Video S2: 5:00 min). The process of ingesting *Teleaulax* was initiated within a period of jumping and attachment of the peduncle (Figure 5I,J, Video S2: 11:48 min), which at times was faintly visible in apical position as a narrow short tube of ca. 2 µm length and 1 µm width (Figure 5K,L). During feeding the volume of the *Teleaulax* prey cell continuously decreased and a flow of intracellular material into the *Prorocentrum* cells was at times visible (Video S2: 16:51 min). A feeding event lasted for approximately 6 min (mean 6 ± 0.08 min, range 2.8–8.1 min, n = 9) and typically ended with a release of one or two small residual granules. Time between two feeding events could be as short as 20 s. Two long-term observations revealed four or five ingestions in 30 or 32 min, respectively. Ingested material from the *Teleaulax* prey cells was clearly visible inside the *P. cordatum* cells with fluorescence microscopy by the distinct yellow fluorescence (Figure 5M–P), and multiple food vacuoles within one predator cell were regularly observed (Figure 5O,P).

### 3.4. Modelling

**Figure 6:**
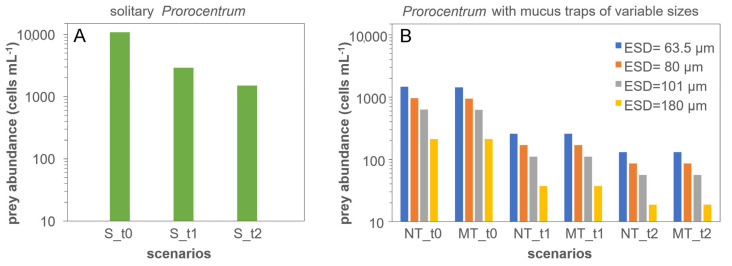
Simulation results showing prey abundances (prey cells mL^−1^) required for *Prorocentrum* to attain satiation growing at 0.6 d^−1^ under different levels of turbulence. (**A**), solitary (S) *Prorocentrum* cells with an ESD of 13 µm. (**B**), *Prorocentrum* cells with non-motile (NT) or motile (MT) mucus traps of different sizes (ESD of 63.5 µm–180 µm). t0, no turbulence; t1, turbulence = 0.0005 m s^−1^; t2, turbulence = 0.005 m s^−1^. Note the log scale of the y-axis.

The results from the in silico investigations are presented in Figure 6. The outputs show that a solitary *Prorocentrum* cell requires an order of magnitude higher prey abundance compared to one with a trap (Figure 6A vs. Figure 6B) to support a given growth rate (0.6 d^−1^) when acquiring all the nitrogen and phosphorus needs via phagotrophy. The mucus trap thus appears to provide a clear advantage enabling the *Prorocentrum* cell to reach satiation with far lower prey cell abundance (Figure 6B).

Turbulence enhances prey capture rates for all *Prorocentrum* cell states (i.e., solitary vs. with traps) with capture, and thence satiation, achieved at lower prey abundances. Thus, the advantage of deploying a trap was amplified under these conditions (ca. 17-fold difference; Figure 6A vs. Figure 6B). For the cells deploying traps, the required prey abundance in the environment decreased pro rata as the volume of the mucus trap (and thence its ESD) increased, resulting in satiation occurring at extremely low prey abundance (e.g., 46 prey cells mL^−1^ at turbulence 0.0005 m s^−1^; Figure 6B). In contrast, motility of the traps does not appear to have any extra advantage; cells with non-motile versus motile traps achieved satiation at similar prey abundance under a given turbulence.

## 4. Discussion

In this study we evidence that the newly described *P. pervagatum*, as well as the ubiquitous and well-studied *P. cordatum* (including various representative strains from both the northern and southern hemispheres), are constitutive mixoplankton. We also show that these species construct mucus traps that immobilize prey, aiding their capture and consumption by peduncular feeding. The production of mucus traps substantially increases the encounter, capture, and ingestion rates of the predatory *Prorocentrum* cells, greatly decreasing the prey abundance required to satiate nutritional needs.

### 4.1. Production and Deployment of Mucus Traps

**Figure 7:**
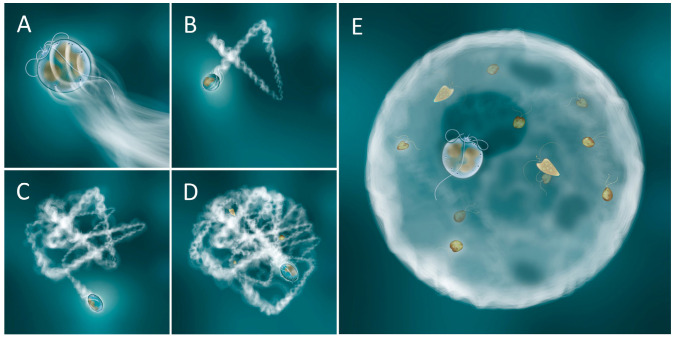
Suggested sequence of events leading to the formation of the mucus trap by *Prorocentrum pervagatum*. (**A**) Solitary cells (cell length ca. 13µm) swim in a spiralling style, releasing mucus from the large pores. (**B**–**D**) Under certain conditions, most likely of very low turbulence, the spiral swimming style coupled with random changes in direction lead to a process of mucus deposition and physical pushing by the *Prorocentrum* cell that leads to the formation of a hollow mucus ball. As the mucus ball takes shape other particles, and notably motile potential prey, become stuck to the outside surface. Towards completion of the trap, with the *Prorocentrum* now located within the hollow ball near one of the few remaining openings, the continuing rotational swimming of the dinoflagellate draws water and particles in through the large opening (a “rotating position”), with water exiting through the porous mucus walls, leaving particles trapped on the inside of mucus ball. Trapped prey are immobilized by a combination of chemical (allelochemical) and physical (mucus threads) means, and may then be consumed (**E**). When the trap is vacated, the decaying remnants then sink, while the *Prorocentrum* builds another trap.

The construction of the trap by *Prorocentrum* is extremely difficult to follow, as the trap only becomes visible once it is already substantial enough to trap particles. Figure 7 presents a schematic of how we envisage the production of the mucus trap to proceed. Both *Prorocentrum* species studied here and *P.* cf. *balticum* have a number of conspicuously large pores (Figure S2) from which we assume the mucus for trap formation is extruded. Using a motion similar to that seen in *P.* cf. *balticum* [9], the cell appears to combine a combination of rotation swimming paths coupled with random re-directional jumps, while extruding mucus, to produce a hollow spherical structure of approximately 100 µm diameter. The jumps result in a new direction for mucus extrusion, and also in the cell bumping into the previously laid mucus, pushing the developing walls of the trap outwards. Over time this repeated random motion creates what could be envisaged as a hollow string ball, but one that also retains at least one, or perhaps two or more, openings large enough for the *Prorocentrum* cell to maintain access to the surroundings, and that is eventually used as an escape route for the trap-producing cell. In the completed trap, the *Prorocentrum* cell often appears to be located, rotating, within one of those gaps (the “rotating position” noted in Section 3, Results; Figure 7E). This continuing rotational motion generates eddies drawing in particles from the surrounding water. Water exits through other gaps, though there appears to be sufficient porosity across the entire trap to allow water to flow through the trap wall, but sufficient obstruction from mucus threads to entrap particles.

Once constructed, the mucus traps effectively immobilize and retain particles down to at least bacteria sizes. Bacteria and microalgal prey adhere to both the exterior (upon contact with the mucoid surface) and interior (after being drawn in through eddies created by the rotating *Prorocentrum* cell) surfaces. As noted for *P.* cf. *balticum* [9], microalgal prey within mucus traps produced by *P. pervatagum* and *P. cordatum* were immobilized, but not killed. This is suggestive of being physically entrapped and perhaps partly narcotized. The observation that *Teleaulax* is significantly immobilized shortly after addition to a dense *Prorocentrum* culture even without being trapped (Video S1: 9:40 min) suggests that allelochemicals may also be involved, as suggested to be deployed by other predatory dinoflagellates [18,19,20]. There is also a possibility that potential prey is attracted by chemo-taxis to dissolved organics excreted from the *Prorocentrum* cell [9], from other trapped organisms, or from decaying remnants of predated organisms. Either way, the mucus trap physically disables the potential prey which aids subsequent consumption.

### 4.2. Phagotrophic Feeding Mechanism in Prorocentrum

Ingestion of prey biomass occurs via a peduncle feeding mechanism, through which the contents of the prey are sucked out (Figure 2 and Figure 5I–L). Both *P. pervagatum* and *P. cordatum* use this device, similar to that observed in *P.* cf. *balticum* [9]. That peduncle feeding for *P. cordatum* has not been observed before is surprising since photo-phago-mixotrophy in this common species was first reported almost 30 years ago [21], with this species being the subject of many further studies [7,8,22,23,24]. More recently, work has focused on various cytological and nutritional aspects of *P. cordatum* [24,25,26,27,28,29,30], with a recent review describing the fate of prey as “captured in the flagellar canal, the prey is engulfed by *Prorocentrum* using receptormediated endocytosis or micropinocytosis” [31]. Such misconceptions underline the importance and usefulness of traditional light microscopy observations to better unveil cellular mechanisms of phago-mixotrophy of protistan species.

Prior to the direct observations of *Prorocentrum* peduncular feeding by Larsson et al. (2022), and in the present study, Transmission Electron Microscopy (TEM) of the intracellular microtubular baskets within various species of *Prorocentrum* [32,33] including *P. cordatum* [29,34] suggested the capability of peduncular feeding in this genus. More observations are required to confirm if direct engulfment, as described by Jeong et al. [7] for four planktonic *Prorocentrum* species, and thus multiple feeding mechanisms, exists within the Prorocentrales (similar to the diversity in *Karlodinium* [35]), or if peduncle feeding is the predominant form also for other species of *Prorocentrum*. Accordingly, for our in silico investigations we assumed that some *Prorocentrum* cells are able to feed without using a mucus trap to physically restrain their prey, though it is not clear from our work that such an event can actually occur for *P. pervagatum* and *P. cordatum*.

The accessory pore of *Prorocentrum*, which is located in the apical region next to the larger flagellar pore (Figure S2), and for which no function is conclusively documented, may be the exit site for the peduncle. For dinoflagellate species where the peduncle emergens area has been studied with TEM sectioning, there is a special structure where that is not associated with the flagella pore(s) [36,37]. If the accessory pore is indeed the exit site for a peduncle, then it would be especially interesting to explore if those species of *Prorocentrum*, where the diameter of the accessory pore is drastically reduced (i.e., the *P. triestinum* complex of *P. triestinum* and *P. redfieldii,* see [38]) are also mixoplanktonic, and if so, which feeding mechanism they use.

Even with a prey item immobilized in the trap, the successful attachment of the peduncle by *Prorocentrum* can take several attempts, and the subsequent process of prey content extraction takes quite some time (ca. 6 min). This is the same duration reported for *Prorocentrum shikokuense* to ingest a comparably sized cryptophyte [7], suggesting that this related species probably also uses a peduncle for feeding. It is highly likely that for small species of *Prorocentrum,* peduncular feeding upon (especially motile) prey is necessarily linked to mucus-trap production to restrain the prey. Coincidentally, that trap also greatly aids in feeding at low prey abundances (Figure 6).

In peduncular feeding the contents of the prey (excluding certain components and the cell exterior) are drawn into a vacuole for digestion. Whether the predator exudes enzymes through the peduncle for initial digestion of material in the prey to fluidise the contents is not known. However, one may expect digestion of material within the *Prorocentrum* vacuole to proceed rapidly with each feeding event, producing what amounts to a bag of digestate within a vacuole. Evidence for this assumption (i.e., 1 feeding event = 1 food vacuole), on which ingestion rate calculations via counting the number of food vacuoles were based, comes from direct observations (Figure 2F–L). Thus, for *P. pervagatum* only one food vacuole was formed per feeding event, and a cell with one food vacuole was observed to acquire a second food vacuole after the next feeding event.

### 4.3. Advantages for Phagotrophy through Trap Deployment

The rates of feeding we report for *P. pervagatum* (approximately 10–12 prey d^−1^, Figure 4G,H) are considerably higher than the rates (1 prey d^−1^) reported for *P.* cf. *balticum* [9]. This could be explained by absolute and/or relative differences in prey and predator size, and in their growth conditions. It could equally reflect intrinsic differences in the evolution of these species, and their preferred prey in different natural environments. The main driver for phagotrophy varies across different mixoplankton species, and indeed within species of the same genus, between provision of nutrients (e.g., nitrogen, phosphorus versus carbon; [39]). Feeding may or may not enhance growth above that possible by phototrophy alone [40]; it is important to recall that phagotrophic feeding is the ancient trait, and not one acquired by phototrophic protists [41,42]. Feeding by *Prorocentrum* was observed only after the construction of a mucus trap and when prey cells were immobilized, though the production of a mucus trap is an active and likely energetically expensive process (Figure 7). All phototrophs leak significant amounts of photosynthate (often assumed to be around 10–20%); the production of a mucus trap would at the least provide potential for the recovery of such a loss. In addition, the acquisition of nutrients in the form of prey digestate would negates the costs of assembling amino acids and notably of the extreme cost (equating to ca. 20–30% of concurrent C-fixation) in assimilating nitrate [43].

Another cost/risk that could be negated through deployment of mucus traps is that associated with motility. A *Prorocentrum* cell within a trap can no longer move rapidly. On one hand this is advantageous as movement typically attracts predators, while on the other hand the cell is unable to optimise its position in the water column for light and acquisition of inorganic nutrient. Our model calculations (Figure 6) indicate a significant advantage of deploying a mucus trap; this is aside from the advantage, or indeed the necessity, in using the trap to restrain the prey to permit peduncular feeding. The motion of the *Prorocentrum* within the trap, drawing water and prey in, must impart some level of trap motility. However, this is difficult to examine under the microscope because slow motions of a trap are difficult to separate from unavoidable convergence flows in the illuminated observation chamber. However, the size of the trap, plus the motility of the prey, means that any advantage of the trap actually moving is minor (Figure 6).

The eddy currents generated by a *Prorocentrum* cell within a trap must, however, impart a degree of relative motion between potential prey and the trap. Even very low turbulence levels greatly increase encounter rates between prey and the traps, although from the culture experiments (Figure 3D–F; decreased ingestion when samples were frequently mixed) it is clear that trap formation and operation is disturbed by turbulence. This may be a consequence of a physical disturbance to the trap initiating a predator-avoidance response, or it may simply disrupt the integrity of the trap structure. The latter event is perhaps more likely during early stages of trap construction, especially if that depends on the random motion pathways of the cell leading to the formation of the mucus sphere (Figure 7).

### 4.4. Mucus Structures Constructed by Dinoflagellates

Mucus production by dinoflagellates is not uncommon, with many species such as *Gonyaulax hyalina* [44], *G. fragilis* [45], and *Lepidodinium chlorophorum* [46] known to develop gelatinous planktonic blooms [47]. Mucoid material may be extruded via extrusomes, of which there are two different basic types—trichocysts and mucocysts [48]. Trichocysts are organelles that discharge long and distinct threads of variable thickness, which are square in section and have regular transverse banding [49]. Extruded trichocyst threads are visible under light microscopy [50] and thus are likely not involved in mucus-trap formation. Mucocysts are vesicles containing an amorphous granular material [48] and are located beneath the thecal pores of dinoflagellates [51,52]. Moreover, in nearly all *Prorocentrum* species investigated so far, a large number of slightly different mucocysts containing diffuse fibrous material are present in the apical region of the cell beneath the periflagellar area [52,53]. It has been suggested that the pusule, a specialized vacuole with an opening through to the cell surface [54], may be involved in mucilage production [51,55]; the apical location of the pusule in *Prorocentrum* may discharge mucus (or particulate matter) through the accessory pore [55,56]. Regularly shaped and smooth hyaline mucoid sheaths around dividing cells or chains of cells (temporary division cysts) [57,58,59], the production of irregular mucous stalk-like structures at the apical cell end [59], or large amounts of mucus that adhere multiple cells as colonies on substrates [56,60] have been reported for certain benthic *Prorocentrum* species.

The chemical composition of the mucus trap produced by *P. pervagatum* and *P. cordatum* is unknown, but it is likely to be similar to that of *P.* cf. *balticum*, where positive staining of mucus traps with the acid polysaccharide stain Alcian Blue and the protein stain Coomassie Brilliant Blue suggest a composition analogous to transparent exopolymeric particles [9]. In the search for possible structural prerequisites for mucus extrusion during trap construction it is notable that for most species of the *P. cordatum* clade the presence of two size classes of thecal pores [10] are reported, with large pores having holes with a diameter of ca. 0.3 µm. Moreover, all three species shown to produce mucus traps share the presence of a distinct, short row of mostly three large pores in apical position on the right thecal plate ([9]; see also Figure S2). Therefore, it may be speculated that large pores with mucocysts are involved in mucus release during trap construction. However, more ultrastructural TEM studies on the location and type of extrusomes of trap-forming species, and/or direct microscopic observations of trap formation, are needed to fully elucidate the structural and behavioural basis of mucus trap construction in *Prorocentrum*.

Utilizing mucus to catch prey is not unique to *Prorocentrum* but has also been recorded for a number of other phagotrophic dinoflagellate species [61]. Prey of the large and voracious grazer *Noctiluca scintillans* attach or become embedded in strings or clumps of mucus before ingestion [62]. Species of the benthic gonyalacalean mixoplankton genus *Ostreopsis* [63,64] produce large amounts of mucilage. Detailed observations have revealed that cells of *O.* cf. *ovata* extrude mucoid compounds and co-operate in rapid formation of larger mucus strings. This mucoid network, likely enriched with toxins (palytoxin-like compounds, [47]), entraps small organisms that are subsequently approached and attacked by *Ostreopsis;* it has been suggested that this “spider strategy” serves as a feeding aid [65,66]. In contrast to the delicate mucus traps of *Prorocentrum*, *Ostreopsis* mucilage has a complex and rigid structure made from a network of long trichocysts fibres in an amorphous matrix of acidic polysaccharides [47]. Various species of planktonic *Dinophysis* also produce mucus either on the cell surface [67] or as free-floating clumps in which the prey *Mesodinium rubrum* is trapped and immobilized before peduncular ingestion [19,68]. It has been suggested that toxic compounds imbedded in the mucus from *Dinophysis* help immobilize prey [19], a method akin to that deployed by *Ostropsis*; the agent that narcotises the prey of *Prorocentrum*, as we observed, is unknown.

A mucus trap very similar to that produced by *Prorocentrum* is also produced by the gonyaulacalean species *Alexandrium pseudogonyaulax* [18,69]. Similarities with the results we report for *Prorocentrum* include: (1) the matrix of the *A. pseudogonyaulax* trap is also described as “invisible” in the absence of captured prey particles [69]; (2) *A. pseudogonyaulax* cells are attached to the mucus trap by the tip of the longitudinal flagellum; (3) *A. pseudogonayaulax* traps are regularly abandoned and accumulate at the bottom of the culture vessel [69]; (4) Motile cells caught in *A. pseudogonyaulax* traps are immobilized with evidence indicating that excreted toxic substances are involved in the feeding strategy [18]. These similarities of mucus-trap-assisted phagotrophy in several evolutionary independent lineages indicate that this may be an advantageous strategy occurring more broadly than currently appreciated, and highlights the need for more detailed investigations of other dinoflagellate groups.

### 4.5. Is Mucus-Trap-Assisted Phagomixotrophy More Widespread in Prorocentrum?

**Figure 8:**
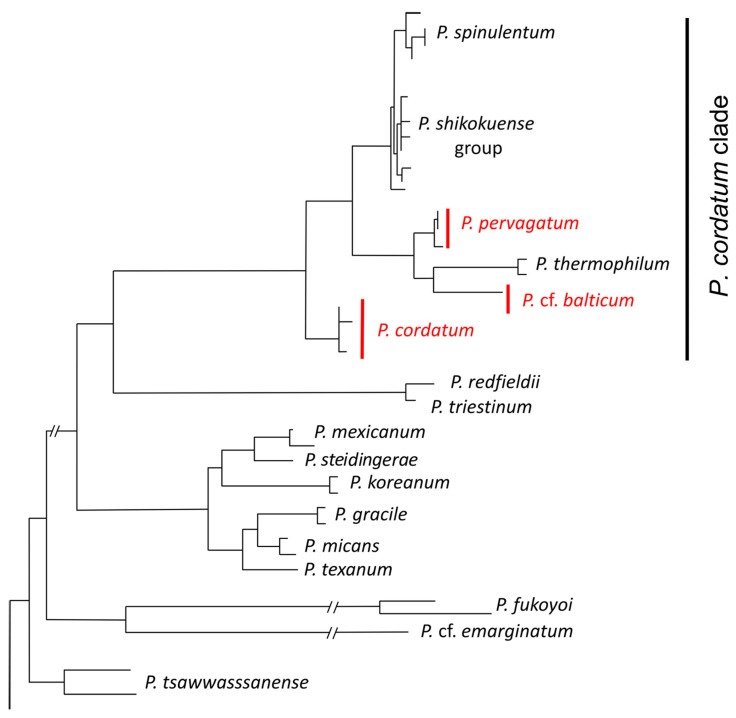
All three *Prorocentrum* yet reported to produce mucus traps (in red) are members of the *Prorocentrum cordatum* clade. Schematic cutout of a phylogenetic tree based on concatenated rRNA sequences, redrawn from [10].

Together with the study of Larsson et al. [9], mucus-trap formation is now documented for three species of small planktonic *Prorocentrum—P.* cf. *balticum*, *P. cordatum*, and *P. pervagatum*. All three species are members of one evolutionary lineage forming a statistically well-supported clade of small species of *Prorocentrum* ([10]; Figure 8). This so-called *P. cordatum* clade (based on the first named species of this group) encompass small planktonic species with numerous small projections (knobs, spines) on the plate surface. It may be hypothesized that phagotrophy assisted by mucus traps will also exist in other species within this cluster (i.e., *P. spinulentum*, *P. shikokunese*, and *P. thermophilum*). Phagotrophy in *P. shikokuense* (reported as its junior synonym *P. donghaiense*) has been described by Jeong et al. [7], and is further indirectly supported by the observation that under N-starvation, genes involved in endocytosis and phagosomes are upregulated [70]. While the use of mucus traps was not documented, Jeong et al. [7] reported that all *Prorocentrum* spp. (including *P. shikokuense*) fed by engulfing the prey cell through the suture at multiple locations around the cell. While micrographs evidence such a feeding mode for the larger species *P. micans* [7], little evidence is reported for *P. shikokuese,* and therefore mucus-trap-assisted mixotrophy via peduncle feeding may have been overlooked. The other species within the clade, *P. spinulentum* and *P. thermophilum*, were only recently described [10,13] and remain poorly studied. Preliminary observations on the type strain of *P. spinulentum* 1-D3 (U. Tillmann, unpublished data) failed to identify mucus trap production. However, this strain has been grown in monoculture since isolation in 2018 and thus may have lost its phagotrophic capability, as has been shown to occur with mucus-trap formation of *Alexandrium pseudogonyaulax* [71]. More detailed observational studies on multiple and newly established strains of *Prorocentrum* spp. are needed to evaluate if mucus-trap formation is widespread and a common trait among the species of the *P. cordatum* clade, or indeed in other clades of planktonic *Prorocentrum*.

## 5. Conclusions

The production of mucus traps appears to be an adaptation employed by multiple members of one major lineage of planktonic *Prorocentrum* species. Trap formation demonstrates coupling of the exudation of mucus with peculiarities of the rotational and jumping motion of a *Prorocentrum* cell collectively leading to the formation of a “hollow string ball”-like structure inhabited by its creator. The trap provides a means to significantly increase the encounter with potential prey items, and perhaps provides a critical advantage (necessity) in immobilizing those prey cells to enable deployment of the peduncular feeding mechanism. Trap construction and deployment appears optimised for growth in waters of low turbulence, likely where inorganic nutrients are, or have become, limiting and hence there are prey available. That the mechanism has remained concealed from science for so long (noting that a few other dinoflagellates have also been recorded to use mucus; [18]) likely reflects that no one was carefully looking for this. The common assumption that phototrophic dinoflagellates are “phytoplankton”, rather than mixoplankton, and the common practices of swirling culture flasks (which would destroy any mucus traps) and maintaining organisms in “uni-algal” suspension (which can eventually select against expression of phagotrophy; [71]) would have collectively prevented attention being drawn to such mucus trap formation events. How all of this may play out in an ecological setting is yet to be determined.

## Figures and Tables

**Table 1:** Information on strains and culture conditions. Temp = Temperature [°C]; Sal = Salinity; PFD = photon flux density (µmol m^−2^ s^−1^; L:D = light:dark cycle [h:h]); NORCCA = Norwegian Culture Collection of Algae; KAC = Kalmar Culture Collection; ANACC = Australian National Algae Culture Collection. n.a. = not available.

Species	Strain	Reference	Isolator Date	Origin	Growth Conditions				
					Temp	Sal	PFD	L:D	Medium
*P. pervagatum*	PM-01	[12]	U.Tillmann 2017	Labrador Sea	15	33	80	16:8	1/10 K
*P. pervagatum*	LP-D3	[12]	U.Tillmann 2020	North Sea, off Denmark	20	33	80	16:8	1/10 K
*P. pervagatum*	LP-D10	[12]	U.Tillmann 2020	North Sea, off Denmark	20	33	80	16:8	1/10 K
*P. cordatum*	BS 4-A5	this study	U.Tillmann 2021	southwestern Black Sea	20	20	80	16:8	1/10 K
*P. cordatum*	BS 4-B5	this study	U.Tillmann 2021	southwestern Black Sea	20	20	80	16:8	1/10 K
*P. cordatum*	BS 4-G2	this study	U.Tillmann 2021	southwestern Black Sea	20	20	80	16:8	1/10 K
*P. cordatum*	DWER-PM23A8	this study	M.Larsson 2021	Wilson Inlet, Great Southern Region	23	20	80	12:12	K
*P. cordatum*	DWERP-M23H9	this study	M.Larsson 2021	Wilson Inlet, Great Southern Region	23	20	80	12:12	K
*P. cordatum*	DWER-PM19E6	this study	M.Larsson 2021	Murray River, Peel Region	23	25	80	12:12	K
*P. cordatum*	DWER-PM19F8	this study	M.Larsson 2021	Murray River, Peel Region	23	25	80	12:12	K
*Teleaulax* *amphioxeia*	k-1837	NORCCA	n.a.	n.a.	15/20	20/33	80	16:8	1/10 K
*Rhodomonas* *salina*	KAC-30	KAC	n.a.	n.a.	15/20	33	80	16:8	1/10 K
*Rhodomomas* *salina*	CS-24	ANACC	n.a	n.a.	23	20/25	80	12:12	K

**Table 2:** Constant parameter values in the modified SCEB model describing the encounter and feeding of the predator *Prorocentrum*. ^a^, Flynn and Mitra [17] and references therein; *, indicates values derived from observational and/or experimental data presented in this work.

Parameter	Definition	Unit	Value
*AE*	assimilation efficiency of the predator	dimensionless	0.8 ^a^
*Cap_pot_*	likelihood of a *Prorocentrum* cell (single or with mucus trap) capturing encountered prey	dimensionless	0.2 ^a^
*Cap_max_*	maximum prey cells that can be captured daily by a *Prorocentrum* cell (solitary or within a mucus trap)	prey predator^−1^ d^−1^	12
*ESD_prey_*	equivalent spherical diameter of prey cell	µm	8 *
${ESD}_{pred}^{cell}$	equivalent spherical diameter of predator cell	µm	13 *
${ESD}_{pred}^{trap}$	equivalent spherical diameter of trap	µm	80–180 *
*sw_Mot_*	Scalar for motility of predator calculated from swimming speeds	dimensionless	0.7–1.3 *
*µ_max_*	maximum growth rate of *Prorocentrum*	gC (gC)^−1^ d^−1^	0.6 *
*w*	turbulence	m s^−1^	0–0.001 ^a^
*N*	prey abundance	number m^−3^	user selected

## Data Availability

Data presented in this study are in the article and Supplementary Materials.

## Supplementary Materials

The following supporting information can be downloaded at: https://www.mdpi.com/article/10.3390/microorganisms11071730/s1, Video S1: Video documentation *P. pervagatum*. Video S2: Video documentation *P. cordatum.* Figure S1: *P. cordatum* from western Australia; Figure S2: Scanning Electron Microscopy micrographs.
