# Supplementary figures and images for: Mucus-Trap-Assisted Feeding Is a Common Strategy of the Small Mixoplanktonic Prorocentrum pervagatum and P. cordatum (Prorocentrales, Dinophyceae)
Source: Microorganisms. 2023 Jul 1;11(7):1730. doi: 10.3390/microorganisms11071730 (PMC10384473; doi:10.3390/microorganisms11071730)

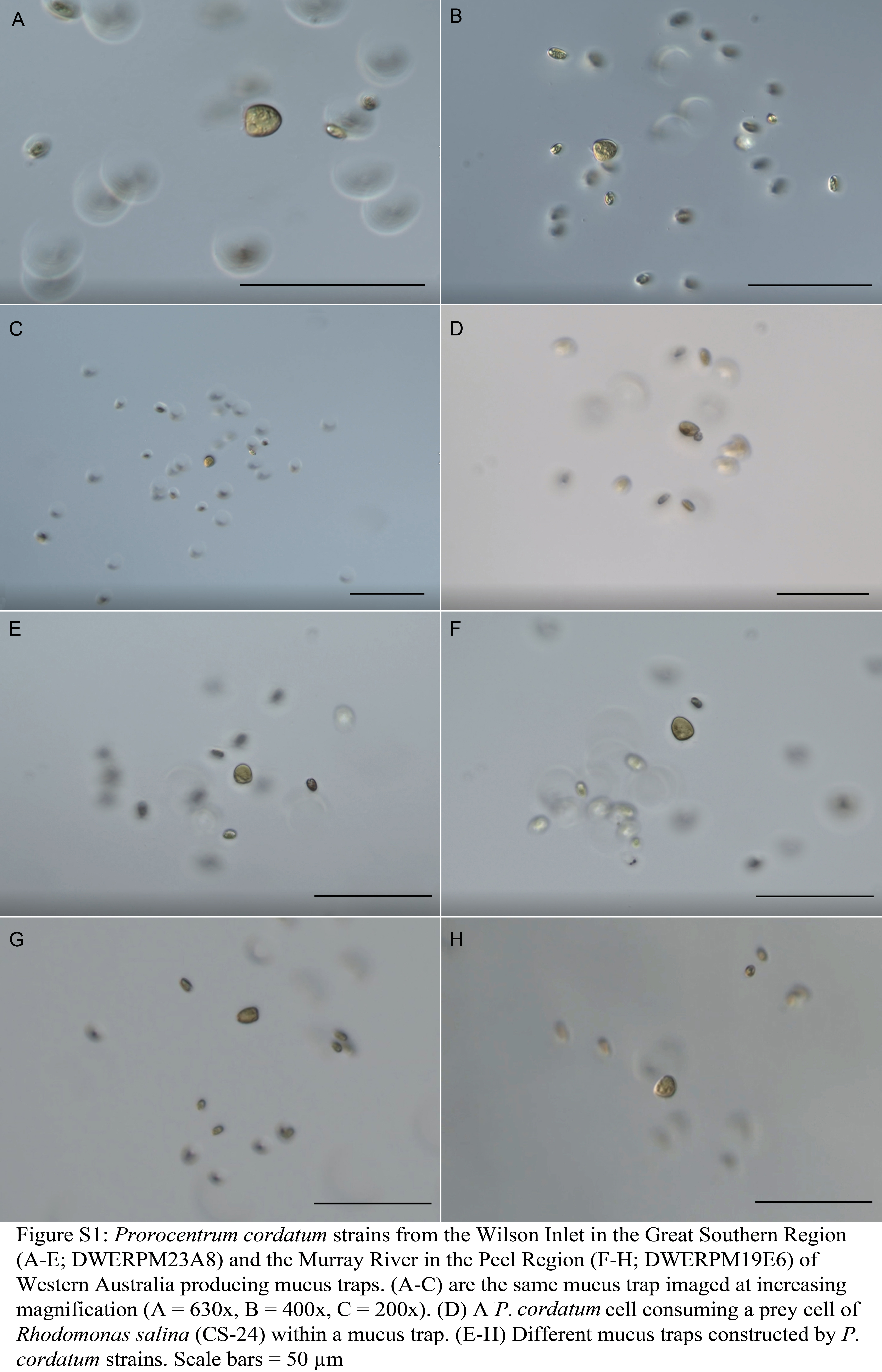

Supplement: Supplementary file 1 [file microorganisms-11-01730-s001.zip › microorganisms-2445040 suppl/Fig_S1.jpg]

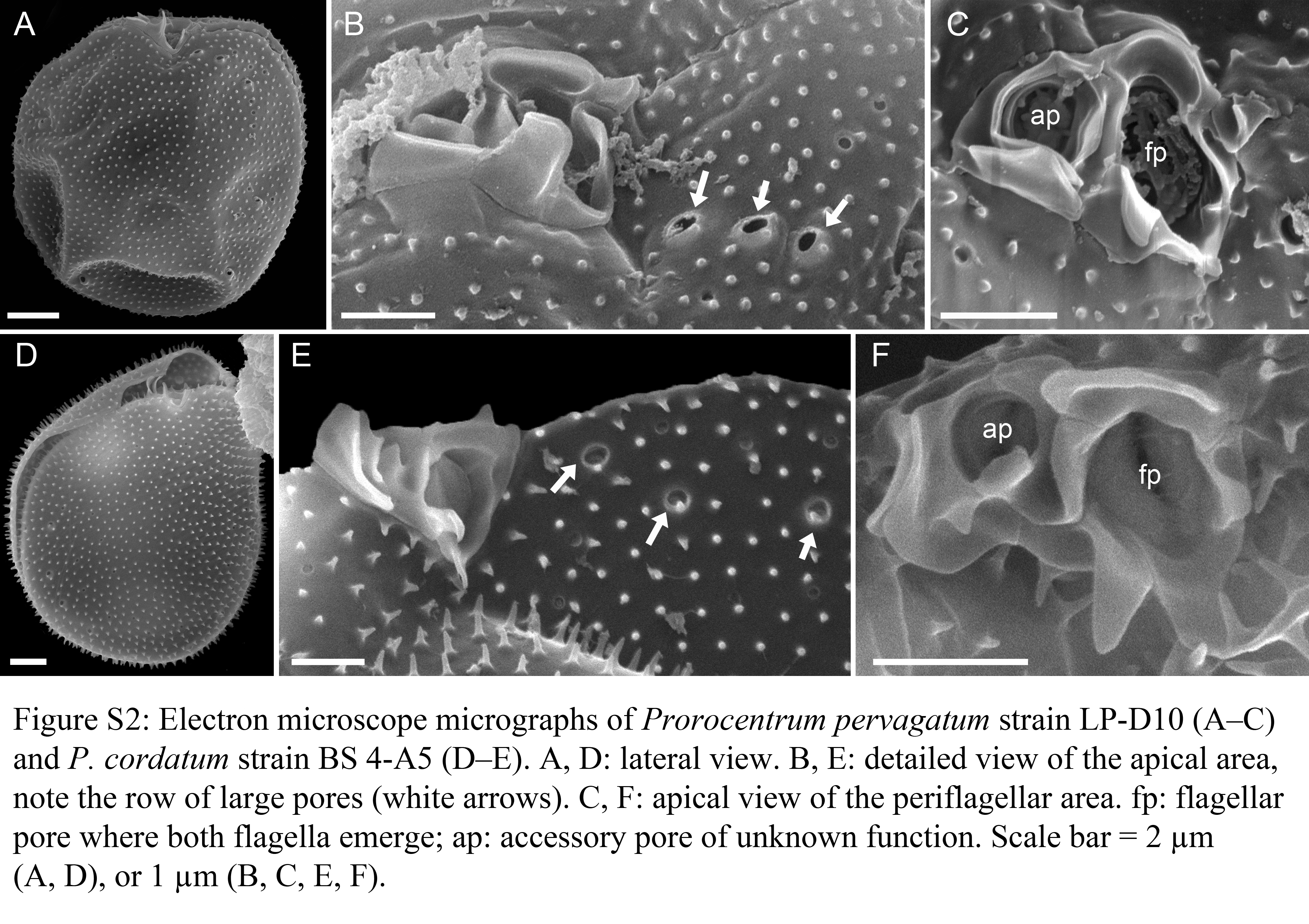

Supplement: Supplementary file 1 [file microorganisms-11-01730-s001.zip › microorganisms-2445040 suppl/Fig_S2.jpg]
